# Overexpression of Pleckstrin Homology Domain-Containing Family A Member 4 Is Correlated with Poor Prognostic Outcomes and Immune Infiltration in Lower-Grade Glioma

**DOI:** 10.1155/2022/1292648

**Published:** 2022-11-11

**Authors:** Baojun Huang, Weijun Pan, Wenchao Wang, Yijian Wang, Pan Liu, Wujun Geng

**Affiliations:** ^1^Department of Anaesthesiology, The First Affiliated Hospital of Wenzhou Medical University, Wenzhou, Zhejiang, China; ^2^Southern Medical University, Guangzhou, Guangdong, China; ^3^Wenzhou Key Laboratory of Perioperative Medicine, Wenzhou, Zhejiang, China

## Abstract

**Introduction:**

The global incidence of brain tumors, the most common of which is lower grade glioma (LGG), remains high. Pleckstrin homology domain-containing family A member 4 (PLEKHA4) has been reported to be related to tumor invasion and growth. However, its role and correlation with immunity in LGG remain elusive.

**Methods:**

We evaluated the expression pattern, prognostic value, biological functions, and immune effects of PLEKHA4 in LGG. We also analyzed the association between PLEKHA4 levels in different tumors, patient prognosis, and its role in tumor immunity. Depending on the type of research data, we used statistical methods such as Student's *t*-tests, Mann–Whitney *U* tests one-way ANOVA tests Kruskal–Wallis tests Pearson's or Spearman's correlation analysis Chi-square and Fisher's exact tests in this paper. *Results and Conclusions*. The results revealed that PLEKHA4 levels were markedly elevated in most tumors (such as LGG). High PLEKHA4 levels are associated with poor overall survival (OS), progression-free interval (PFI) rates, and disease-specific survival (DSS) in LGG patients. Cox regression analysis and nomograms showed that PLEKHA4 levels are independent prognostic factors for LGG patients. According to functional enrichment analysis, PLEKHA4 levels in LGG are associated with immune infiltration and immunotherapy. In conclusion, PLEKHA4 is a potential prognostic marker and immunotherapy target for LGG.

## 1. Introduction

Gliomas, a major malignancy of the CNS (central nervous system), are among the most common brain cancers. There were 83,830 new glioma reports in the US in 2020, and 81,246 patients died of glioma between 2013 and 2017 [1]. Although lower grade gliomas (LGGs) (WHO grades II and III) [2] have better outcomes than grade IV gliomas (glioblastomas (GBMs)), their clinical heterogeneity leads to a high incidence rate and increases the difficulty of treatment [3]. Current diagnostic and treatment methods depend on prognostic factors, such as cancer grade, Karnofsky performance status, initial symptoms, excision scope, cancer dimension along with region, neurological deficits, and certain molecular biomarkers, including 1p/19q codeletion (codeletion of chromosome arms 19q and 1p) [4]. Although the main treatments for LGG, such as surgery, chemotherapy, and radiation therapy, can improve patients' prognoses to some extent, there are some limitations [5, 6].

The common treatment for LGG includes immunotherapy, surgery, and targeted therapies [7, 8]. Immune checkpoint blockers, such as CTLA-4 and PD-1/PD-L1 inhibitors, promote the development of tumor immunological responses for LGG, strengthening the unique role of tumor immune system responses [9–11]. The tumor microenvironment (TME), composed of various blood and stromal cells and immune cells, is crucial for tumor development and progression and depends on the mutual effect among the tumor microenvironment, immune system, and cancer cells [12, 13]. TME contains tumor epithelial cells and supports various cancer cells within a complex dynamic cell population while promoting continuous cell proliferation and invasion, and it is vital for cancer cells to evade immune surveillance [14]. Previous studies have found that TIIC (tumor-infiltrating immune cells) can influence chemotherapy efficiency, prognosis, and immunotherapy efficiency in patients [15]. In addition, there are anticancer and cancer-promoting immune cells in TME, while cancer overwhelming anticancer immune cells might be associated with cancer progression [16]. Therefore, it is critical to assess the immunological features of TME and characterize LGG to identify new biomarkers for predictions and molecules associated with immunity.

PLEKHA4, phosphoinositol 3-phosphate-binding protein 1 (PEPP1), promotes wnt/*β*-catenin signaling-induced G-S transition and proliferation in patients with melanoma [17]. However, PLEKHA4 expression has no relationship with the prognosis of melanomain the previous studies. Moreover, the role of PLEKHA4 in LGG and the effect of PLEKHA4 on LGG immunotherapy is unknown.

This study investigated prognostic and immunotherapeutic markers and their functions in LGG. PLEKHA4 was identified as the gene of interest. Our present study comprehensively analyzed the relationship between the expression levels of PLEKHA4 and prognostic risk of LGG patient and determined the correlations between PLEKHA4 levels and tumor immune infiltrations.

## 2. Materials and Methods

### 2.1. Data Source and Analysis for TCGA Pan-Cancer Analyses

We downloaded the RNA expression profiles and clinical data of LGG patients from TCGA database (https://portal.gdc.cancer.gov/) [2]. We downloaded normal samples from GTEX by UCSC (https://xenabrowser.net/datapages/) due to a lack of normal samples for brain tumors [18]. UCSC processed the sequencing results from the two databases, TCGA and GTEX, and they could be downloaded and used directly. Gene expression profiling datasets (GSE109857, GSE147352, GSE4290, and GSE16011) were retrieved from GEO database (https://www.ncbi.nlm.nih.gov/gds) [19–21]. We downloaded the gene set expression matrix of CGGA-325 and Rembrandt from CGGA (http://www.cgga.org.cn/) [22, 23]. We screened WHO grade 2-3 tumors in these datasets for the next step of analysis based on the definition of LGG. The RNA-sequencing data and clinicopathological characteristics of TCGA pan-cancer were retrieved from UCSC Xena browser. These data were from public databases and did not raise any ethical concerns.

### 2.2. Biological Function, Pathway Annotation, and Gene Set Enrichment Analysis (GSEA)

GSEA was performed to determine if a priori-defined gene set was statistically significant to establish concordant variations between biological states [17]. Gene levels were recognized as phenotype labels. For every analysis, the gene set permutation count was 1,000 times. Pathways with false discovery rates (FDRs) < 0.05 and *p* < 0.05 were significantly enriched.

### 2.3. Analysis of Tumor Immune Signatures

In this analysis, we evaluated (1) the levels of immune checkpoints and the human leukocyte antigen (HLA) gene family [24, 25]; (2) infiltration of stromal and immune cells and the survival among PLEKHA4 high and low subgroups by MCP, CIBERSORT-ABS, and xCell algorithms [26–28]; the results of which can be obtained from TIMER2.0 website (http://timer.comp-genomics.org/); (3) the score of the immune, stromal, estimate, and tumor purity in tumor samples, which was based on R package “ESTIMATE.”; and (4) the association of PLEKHA4 with lymphocytes, immunomodulators, and chemokines in patients with LGG through TISIDB database [29].

### 2.4. Survival and Other Statistical Analysis

Based on correlations between patient survival and gene expression in TCGA/CGGA/Rembrandt sets, the optimal threshold for gene expression (z score normalized data) was evaluated by “survminer” in R. Cutoff values for the training set were used in the other datasets for group categorization. OS analyses were conducted by log-rank tests and Kaplan-Meier (K-M) analyses. Nomograms and Cox proportional hazard regression models were established using univariate and multivariate analyses. We also conducted a further decision curve analysis (DCA) to check the clinical applicability of the nomogram. A prognostic meta-analysis was performed in R to determine the prognostic significance of PLEKHA4. Then, the fixed effects model calculated a pooled hazard ratio (HR) value. Time-dependent receiver operating characteristic (ROC) curves were used to compare different survival factors. The area under the curve (AUC) was obtained using the “pROC” package in R. Unpaired Student's *t*-tests were used to compare the two groups for normally distributed variables, while Mann–Whitney *U* tests were used for abnormally distributed data. For group comparisons, Kruskal–Wallis and one-way ANOVA tests were used as nonparametric and parametric methods, respectively. Based on the normality of the data, Spearman's or Pearson's correlation analysis was used to assess the association between the two groups.

## 3. Results

### 3.1. PLEKHA4 Levels in Human Tumors

**Figure 1:**
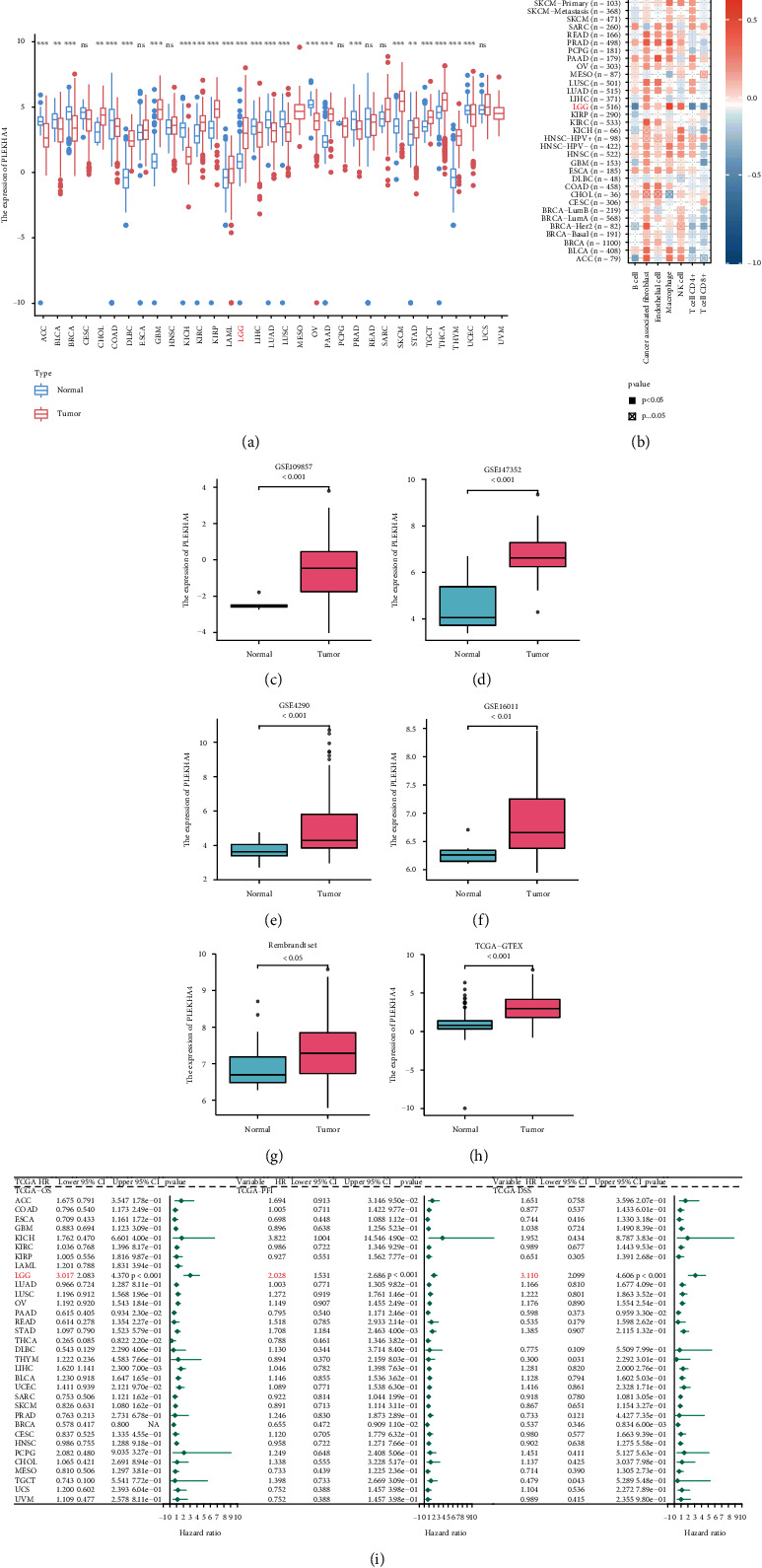
Role of PLEKHA4 in pan-cancer. (a) The mRNA expression of PLEKHA4 between tumour and normal control tissues was assessed from TCGA database.(b) Correlation of PLEKHA4 with the immune-related scores in human solid cancers from TCGA database.(c–h) The PELKHA4 expression levels in LGG and normal brain tissues from the GSE109857, GSE147352, GSE4290, GSE16011, Rembrandt set, and TCGA-GTEX datasets.(i) Univariate Cox regression analysis estimating prognostic value (OS/PFI/DSS) of PLEKHA4 in different cancer types from TCGA database. The length of horizontal line represents the 95% CI for each group. The vertical dotted line represents HR = 0. HR > 1.0 indicates overexpression. PLEKHA4 is an unfavourable prognostic biomarker. ACC: adrenocortical carcinoma; BLCA: bladder urothelial carcinoma; BRCA: breast invasive carcinoma; CESC: cervical squamous cell carcinoma and endocervical adenocarcinoma; CHOL: cholangiocarcinoma; COAD: colon adenocarcinoma; DLBC: lymphoid neoplasm diffuse large B cell lymphoma; ESCA: esophageal carcinoma; GBM: glioblastoma multiforme; HNSC: head and neck squamous cell carcinoma; KICH: kidney chromophobe; KIRC: kidney renal clear cell carcinoma; KIRP: kidney renal papillary cell carcinoma; LAML: acute myeloid leukemia; LGG: brain lower grade glioma; LIHC: liver hepatocellular carcinoma; LUAD: lung adenocarcinoma; LUSC: lung squamous cell carcinoma; MESO: mesothelioma; OV: ovarian serous cystadenocarcinoma; PAAD: pancreatic adenocarcinoma; PCPG: pheochromocytoma and paraganglioma; PRAD: prostate adenocarcinoma; READ: rectum adenocarcinoma; SARC: sarcoma; SKCM: skin cutaneous melanoma; STAD: stomach adenocarcinoma; TGCT: testicular germ cell tumor; THCA: thyroid carcinoma; THYM: thymoma; UCEC: uterine corpus endometrial carcinoma; UCS: uterine carcinosarcoma; UVM: uveal melanoma. ^∗^*p* < 0.05; ^∗∗^*p* < 0.01; ^∗∗∗^*p* < 0.001; ns not significant.

Given the limited number of studies on PLEKHA4 in cancer, we evaluated PLEKHA4 levels in 33 solid tumors, including LGG. First, we used TCGA and GTEx data to compare PLEKHA4 mRNA expression in 33 tumors and normal tissues (Figure 1(a)). PLEKHA4 mRNA levels were significantly higher in most cancers than in normal tissues. We next analyzed the relationship between PLEKHA4 levels and the levels of immune cells and stromal cells in 33 tumors. The results show that PLEKHA4 is closely correlated with the immune microenvironment of multiple cancers, including LGG (Figure 1(b)). To compare PLEKHA4 expression in LGG and normal tissues, we used TCGA-GTEX RNA sequence data (*p* < 0.0001), Rembrandt set (*p* < 0.05), GSE109857 (*p* < 0.001), GSE147352 (*p* < 0.001), GSE4290 (*p* < 0.001), and GSE16011 (*p* < 0.01). PLEKHA4 mRNA levels in LGG tissues were significantly higher than in normal tissues (Figures 1(c)–1(h). Finally, the prognostic significance of PLEKHA4 related to OS, PFI, and DSS was verified using the independent TCGA cancer cohort with 9,163 tumor samples via univariate Cox regression analysis. PLEKHA4 was a prognostic marker in various TCGA cohorts, including cancers with high immunogenicity, immune infiltration, and TMB, including pancreatic adenocarcinoma, breast cancer, thyroid carcinoma, and hepatocellular liver carcinoma (including LGG, Figure 1(i)).

### 3.2. PLEKHA4 Levels in LGG Were Elevated

**Figure 2:**
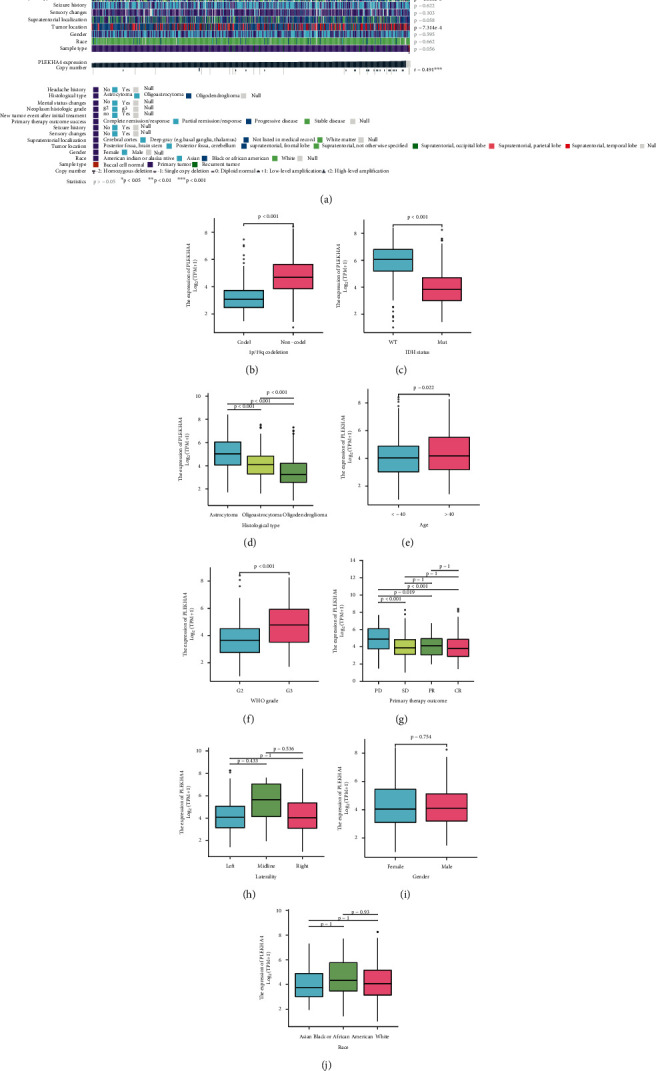
PLEKHA4 expression is elevated in LGG samples from publicly available datasets. (a) Correlations between PLEKHA4 level and clinicopathological characteristics in LGG.(b–j) In the TCGA-LGG dataset, PELKHA4 expression in the different status of 1p/19q codeletion, IDH status, age, gender, WHO grade, laterality, primary therapy outcome, and histological types and race. ^∗^*p* < 0.05; ^∗∗^*p* < 0.01; ^∗∗∗^*p* < 0.001; ns not significant.

First, the role of PLEKHA4 in LGG malignancy was evaluated (Table 1). In MEXPRESS, groups were classified based on various clinical factors, such as age at initial pathologic diagnosis, headache history, histological type, Karnofsky performance score, mental status changes, neoplasm histologic grade, new tumor events after initial treatment, the success of primary therapy, seizure history, sensory changes, supratentorial localization, tumor location, gender, and race. PLEKHA4 displayed a differential expression pattern (Figure 2(a)). According to the 2016 WHO guidelines, 1p/19q codeletion and isocitrate dehydrogenase (IDH) mutations are clinical prognostic markers [30]. Based on the importance of 1p/19q codeletion and IDH mutations in the occurrence and progression of LGG, we compared the expression levels of PLEKHA4 in patients with and without 1p/19q codeletion and IDH mutations. In TCGA dataset, patients with IDH mutations and 1p/19q codeletion had significantly lower PLEKHA4 expression than patients without IDH mutations and 1p/19q codeletions (*p* < 0.001, Figures 2(b) and 2(c)). LGG is divided into several subtypes according to IDH and 1p/19q. 1p/19q-non-codeleted (IDH-mutant or IDH-wildtype) diffuse gliomas are characterized by an astrocytic phenotype, while IDH-mutant and 1p/19q-codeleted cancers are characterized by an oligodendroglial phenotype [31]. The histological classification of LGG was consistent with PLEKHA4 expression (*p* < 0.001, Figure 2(d)). We found that the expression of PLEKHA4 increased with the patient's age and the tumor's grade (all *p* < 0.05, Figures 2(e) and 2(f)). This result was verified in two independent databases, CGGA and Rembrandt datasets (Supplement Figure 1).

Based on RECIST 1.1, assessment criteria for efficacy include PR, CR, SD, and PD. We compared the treatment response of LGG patients and established that patients with elevated PLEKHA4 levels responded poorly to primary treatment, and the disease was in a state of progression (all *p* < 0.05, Figure 2(g)). In addition, we found no difference in PLEKHA4 expression in the tumor site or sex (all *p* > 0.05, Figures 2(h)–2(j)).

### 3.3. Prognostic Significance of PLEKHA4 in LGG

**Figure 3:**
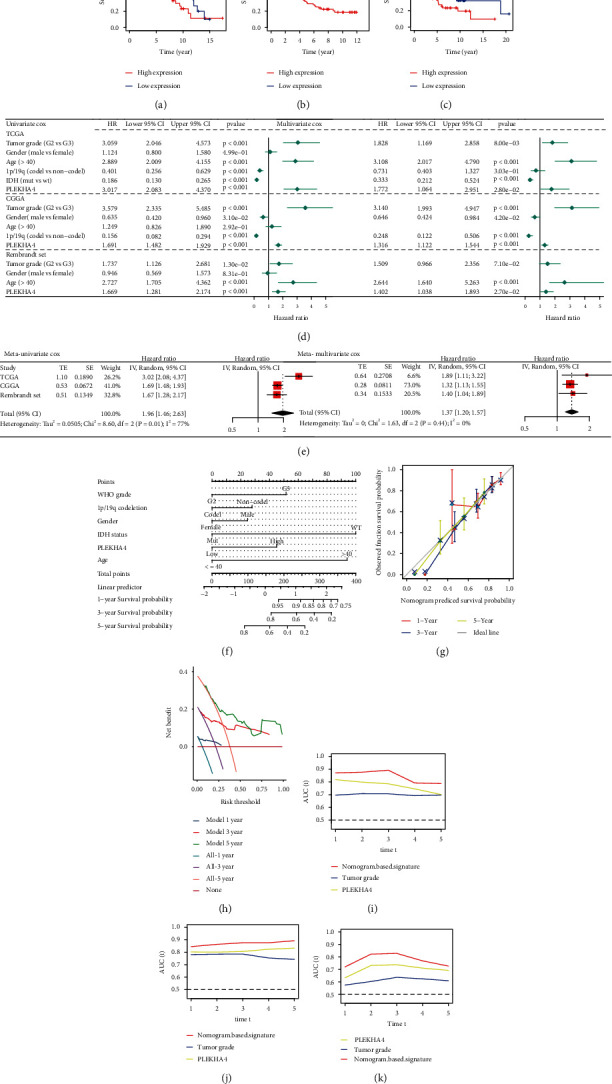
Identification of PELKAH4 as a prognostic gene and construction of a PLEKHA4-based prognostic prediction model.(a–c) OS between PELKHA4 high and low groups in TCGA, CGGA, and Rembrandt datasets. (d) Univariate and multivariate Cox regression analyses of PLEKHA4 level with tumor grade, gender, age, 1p/19q codeletion, and IDH statusin, the TCGA, CGGA, and Rembrandt cohorts. HR and *p* values were displayed. (e) Meta-analysis of prognostic values of PLEKHA4 for patients across three cohorts. A fixed effects model was used to calculate pooled HR value. (f) Nomogram by multivariate Cox regression analysis for predicting the proportion of patients with OS. (g) Plots depict the calibration of model in terms of agreement between predicted and observed OS. Model performance is shown by the plot, relative to the 45-degree line, which represents perfect prediction. (h) The DCA results show that the 1-, 3-, and 5-year net benefit of the nomogram is significantly higher. (i–k) AUC plotted for different durations of OS for nomogram-based signature, PLEKHA4 expression, and tumour stage in the TCGA, CGGA, and Rembrandt datasets. ^∗^*p* < 0.05, ^∗∗^*p* < 0.01, and ^∗∗∗^*p* < 0.001; ns not significant.

To explore the prognostic significance of PLEKHA4 in LGG, we evaluated the relationship between PLEKHA4 mRNA levels and disease outcomes using the Kaplan-Meier plotter. As displayed in Figures 3(a)–3(c), LGG subjects with strong expression of PLEKHA4 showed poorer OS (log-rank *p* = 1.323*e* − 04, 4.619*e* − 14, and 0.008). PLEKHA4 levels were significantly higher in the univariate Cox regression analysis. In addition, PLEKHA4 was established to be an independent prognostic marker in the multivariate Cox proportional hazards regression analyses using TCGA, CGGA, and Rembrandt data (for OS, HR = 1.772, 1.316, and 1.402; 95%CI = 1.064–2.951, 1.122–1.544, and 1.038–1.893; *p* = 0.028, < 0.001, and < 0.001, Figure 3(d)). Then, a prognostic meta-analysis was conducted to elucidate the prognostic significance of PLEKHA4 in all three cohorts. Elevated PLEKHA4 levels were a significant risk factor for OS outcomes (univariate analysis, combined HR = 1.96, 95%CI = 1.28–2.17, and *p* < .001; for multivariate analysis, combined HR = 1.37, 95%CI = 1.20–1.57, and *p* < .001; Figure 3(e)). We built a prognostic nomogram to better predict LGG prognosis by integrating two independent mortality predictors (tumor grade and PLEKHA4 levels) into the multivariate Cox regression model. TCGA data were used to evaluate and verify the nomogram by predicting the 1-, 3-, and 5-year OS outcomes for individual patients (Figure 3(f)). According to the calibration plot, the nomogram better predicted patient OS (Figure 3(g)). The DCA results show that the 1-, 3-, and 5-year net benefit of the nomogram is significantly higher (Figure 3(h)). Concerning OS prediction, the nomogram concordance index was 0.808 in the respective TCGA cohorts. Among the factors evaluated in TCGA, CGGA, and Rembrandt data, AUC values revealed that PLEKHA4 levels and tumor grade best predicted OS (Figures 3(i)–3(k)).

### 3.4. PLEKHA4 Was Correlated with LGG Immune Signature

**Figure 4:**
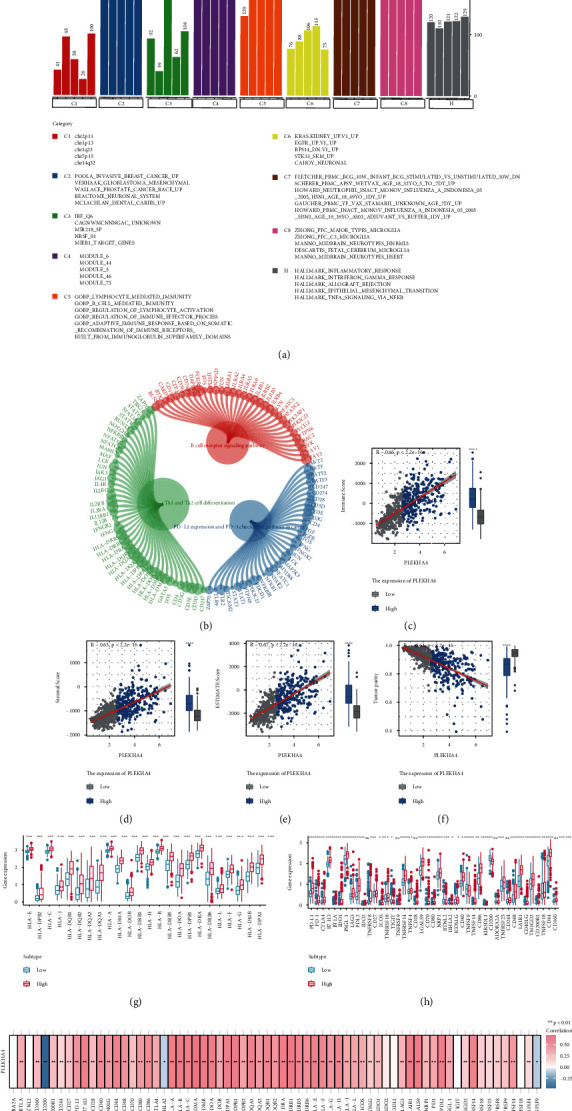
Function enrichment analysis for PLEKHA4 and correlation between PLEKHA4 and expression of the HLA family genes/immune checkpoints. (a, b) GO annotations, GSEA, and KEGG pathways of PLEKHA4 in LGG cohort. (c–f) Association between immune score, stromal score, estimate score, tumor purity, and the expression of PLEKHA4 in the low- and high-PLEKHA4 groups. (g–h) Analyses for the expression of immune checkpoints and HLA family genes in different PLEKHA4 groups. (i) Correlation analysis for the expression of PLEKHA4 and expression of HLA family genes/immune checkpoints. ^∗^*p* < 0.05, ^∗∗^*p* < 0.01, and ^∗∗∗^*p* < 0.001; ns not significant.

We used gene set enrichment analysis to determine the mechanisms associated with different outcomes in the low- and high-PLEKHA4 groups (GSEA). Pathways that whose |normalized enriched score (NES)|>1 and FDR value < 0.05 are presented in Figures 4(a) and 4(b). GO and KEGG enrichment results suggested that PLEKHA4 participated in various activities, including B cell-mediated immunity, immune effector process regulation, lymphocyte-facilitated immunity, lymphocyte activation regulation, Th1 and Th2 cell differentiation, the B cell receptor signaling pathway, and PD-L1 expression PD-1 checkpoint pathway in cancer. The hallmark gene set outcomes also identified terms correlated with tumors, such as inflammatory response, TNFA signaling via interferon-gamma responses, and NFKB. These results indicate that PLEKHA4 may be related to the tumor inflammatory response and immune processes in LGG. According to GSEA enrichment findings, we used ESTIMATE algorithm to establish the relationship between PLEKHA4 levels and immune, stromal, estimate scores, and tumor purity in LGG. The results in Figures 4(c)–4(f) show that PLEKHA4 expression is closely related to immune (*R* = 0.66, *p* < 0.001), ESTIMATE (*R* = 0.67, *p* < 0.001), and stromal (*R* = 0.63, *p* < 0.001) scores but negatively associated with tumor purity (*R* = −0.64, *p* < 0.001). This result was verified in CGGA and Rembrandt sets (Supplement Figure 2). Then, we evaluated the gene expression of 24 HLA family genes and 43 immune checkpoints between the low- and high-PLEKHA4 groups. The Wilcoxon test showed that 37 immune checkpoints and 24 HLA family genes, including HLA-DRA, CTLA-4, BTLA, PD-L1, PD-1, and B7-H3, were markedly modified in the high-PLEKHA4 group (Figures 4(g)–4(i)). We also explored the association of PLEKHA4 with lymphocytes, immunomodulators, and chemokines in LGG patients through TISIDB (Supplement Figure 3). Supplement Figure 4 showed the survival difference between low- and high-immune cell infiltration among PLEKHA4 high- and low-subgroups, respectively, such as CD4 T cells, CD8 T cells, M macrophages, and B cells.

**Figure 5:**
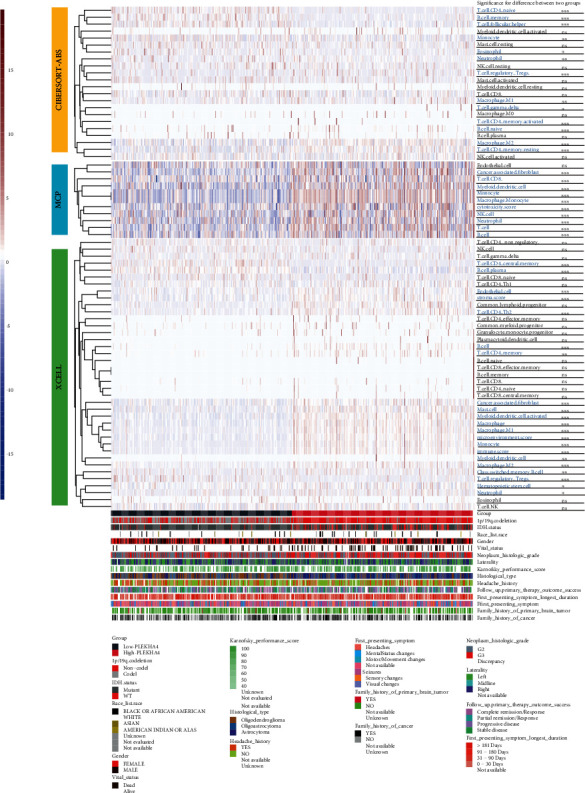
Landscape of immune and stromal cell infiltrations in the low- and high-PLEKHA4 groups. The heatmap shows the normalized scores of immune and stromal cell infiltrations. Blue represents cells with lower infiltration in the high-PLEKHA4 group, and red represents cells with higher infiltration in the high-PLEKHA4 group. The statistical difference between the two groups was compared by the Wilcoxon test. ^∗^*p* < 0.05, ^∗∗^*p* < 0.01, and ^∗∗∗^*p* < 0.001; ns not significant.

Immune cell infiltration levels were assessed using CIBERSORT-ABS, MCP, and xCell between the high- and low-PLEKHA4 groups (Figure 5). Most stromal and immune cells were suppressed in the low-PLEKHA4 group. In addition, CD4+ T cells, cancer-associated fibroblasts, neutrophils, T cell helper 1 (Th1) cells, and memory B cells were highly infiltrated in this group. M2 macrophage infiltration was higher in the high PLEKHA4 group (*p* < 0.05). M2 macrophages promote cancer cell proliferation and angiogenesis while suppressing anticancer immunity. Meanwhile, there were no significant differences in CD8 T cells between the groups (*p* > 0.05).

## 4. Discussion

The tumor microenvironment (TME) is often used as one of the predictive biomarkers and plays an important role in the selection of immune checkpoint inhibitors in tumor patients. TME refers to noncellular and cellular constituents found in and around tumors. Typically, the TME is divided into stromal cells, extracellular matrix (ECM), and immune cells [32]. The ECM comprises laminin, collagen, integrin, fibronectin, glycosaminoglycan, and matrix metalloproteinase (MMP), and secreted cysteine-rich acidic protein. Structural support, biochemical signals, and reagents are used for tumor cell growth [33, 34]. Stromal cells comprising mesenchymal stromal cells, fibroblasts, pericytes, and fat cells secrete various growth factors of various components, such as matrix metalloproteinases and ECM, to enhance tumor cell proliferation and migration [35]. In addition, the immune environment is involved in tumor progression and the overall effectiveness of cancer treatment [36].

The TME primarily suppresses anticancer immunity and promotes tumors. Immunosuppressive effects of the TME are due to immune-modulatory activities of immune cells, including tumor-associated macrophages (TAMs), tumor-infiltrating dendritic cells, neutrophils, and T cell-mediated immune responses [36]. Therefore, the factors involved in the immune regulation of TME were evaluated in this research for tumor immunotherapy.

LGG is one of the most common tumors of the brain and seriously affects health [37]. Its treatment usually requires multiple modalities, including surgery followed by chemoradiotherapy (CRT) and immune checkpoint inhibitors. However, patients often have poor prognoses. Therefore, it is critical to determine new therapeutic targets for LGG. We proposed that PLEKHA4 is a new prognostic target of LGG and studied its biological function and correlations with immune cell infiltration.

As a member of PLEKHAs, PLEKHA4 has always been considered an important factor in ubiquitination in the WNT pathway [38]. However, a study in 2021 found that PLEKHA4 can also play an important role in tumors. PLEKHA4 can accelerate the proliferation of melanoma cells and the transition of the G-S cell cycle. Knockout of PLEKHA4 in nude mice can inhibit the growth of melanoma [17].

We evaluated the role of PLEKHA4 in LGG's TME. We performed a bioinformatic analysis of public data to understand the potential function of PLEKHA4 LGG in detail. First, we downloaded TCGA and GTEX data through USCS to assess PLEKHA4 levels in all normal and tumor tissues and found that PLEKHA4 was elevated in most tumors, such as LGG. The expression difference of PLEKHA4 in LGG has been verified in multiple databases. The tumor immune infiltration and clinical prognostic analyses showed that PLEKHA4 is associated with immune infiltration and prognostic outcomes for various cancers, including LGG. These findings show that PLEKHA4 is elevated in gliomas and is linked to immune cell infiltration and prognostic outcomes in LGG patients. IDH mutations and 1p/19q coding conditions mediate the prognostic outcomes for LGG patients in addition to tumor grade, age, histological type, and other factors [39, 40]. We analyzed the association between PLEKHA4 levels and multiple clinical factors in LGG. The high expression of PLEKHA4 is often accompanied by IDH nonmutation, 1p/19q noncoding, higher tumor grade, and patient age. Patients with higher PLEKHA4 expression have poorer tumor treatment responses. To elucidate the prognostic significance of PLEKHA4 in LGG patients, Kaplan-Meier curves were used to plot differences in the survival of glioma patients with high and low PLEKHA4 expression. Univariate Cox and multivariate Cox regression calculated the prognostic diagnostic value of PLEKHA4. It was found that PLEKHA4 is an independent prognostic factor, which was verified by a prognostic meta-analysis. The subsequent nomogram and ROC curve drawing further illustrated the prognostic diagnostic role of PLEKHA4 in patients with LGG. To better understand the role of PLEKHA4 in LGG, we performed GSEA functional enrichment after grouping according to the high and low expression of PLEKHA4 using the MsigDB gene set. We found that PLEKHA4 levels are associated with infiltration and differentiation of various tumor immune cells and the PD-L1 expression PD-1 checkpoint pathway. Through ESTIMATE calculation, we found that PLEKHA4 levels are closely associated with tumor immune infiltration. The PD-L1, CTLA4, and HLA families are important targets of immunotherapy [41]. As a result, we investigated the relationship between PLEKHA4 and them and discovered that PLEKHA4 expression is closely related to them, implying that PLEKHA4 may be an important target for patient immunotherapy in LGG. We also found an association of PLEKHA4 with lymphocytes, immunomodulators, and chemokines in patients with LGG through TISIDB. Finally, we evaluated the relationship between PLEKHA4 levels and the infiltration of various immune cells using CIBERSORT-ABS, MCP, and XCELL algorithms. PLEKHA4 levels were associated with infiltrations of various cells, including B cells, M2 macrophages, CD4 T cells, and neutrophils.

**Figure 6:**
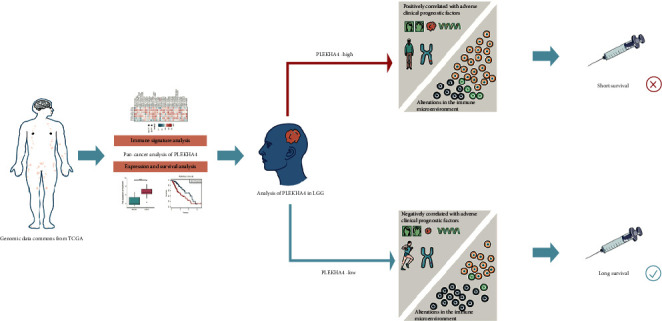
Graphical abstract for workflow and comprehensive characterization of PLEkHA4 in LGG.

In conclusion, the expression of PLEKHA4 is increased in LGG patients. PLEKHA4 levels affect various clinical variables. Increased PLEKHA4 levels reduce survival times in LGG patients with various clinical characteristics and are an independent risk factor for poor prognosis in LGG. Moreover, PLEKHA4 levels were markedly correlated with the infiltration of various immune cells and checkpoints. In conclusion, PLEKHA4 is a potential prognostic marker and therapeutic target for LGG (Figure 6). Studying how PLEKHA4 affects TME will provide immunotherapeutic options for LGG. There are some shortcomings in this manuscript. Due to the particularity of the site of neuronal tumor development and the prognosis of patients, this paper does not conduct single-cell sequencing of public data, which will be used in future research to supplement.

## 5. Conclusion

This study shows a potential correlation between the expression of PLEKHA4, the prognosis of LGG patients, and the tumor immune microenvironment. Based on these findings, we hypothesized that the low expression of PLEKHA4 may benefit LGG patients and their immunotherapy. However, further prospective randomized controlled trials are required to test the efficacy of PLEKHA4 in LGG patients.

## Figures and Tables

**Table 1:** Correlation between PLEKHA4 expression and clinicopathologic factors in the TCGA cohort.

Characteristic	Low expression of PLEKHA4	High expression of PLEKHA4	*p*
*n*	264	264	
WHO grade, *n* (%)			< 0.001
G2	143 (30.6%)	81 (17.3%)	
G3	94 (20.1%)	149 (31.9%)	
IDH status, *n* (%)			< 0.001
WT	13 (2.5%)	84 (16%)	
Mut	249 (47.4%)	179 (34.1%)	
1p/19q codeletion, *n* (%)			< 0.001
Codel	147 (27.8%)	24 (4.5%)	
Noncodel	117 (22.2%)	240 (45.5%)	
Age, *n* (%)			0.433
< =40	137 (25.9%)	127 (24.1%)	
> 40	127 (24.1%)	137 (25.9%)	
Primary therapy outcome, *n* (%)			0.002
PD	38 (8.3%)	72 (15.7%)	
SD	78 (17%)	68 (14.8%)	
PR	31 (6.8%)	33 (7.2%)	
CR	80 (17.5%)	58 (12.7%)	
Gender, *n* (%)			0.484
Female	124 (23.5%)	115 (21.8%)	
Male	140 (26.5%)	149 (28.2%)	
Age, median (IQR)	40 (33, 52)	41 (32, 54.25)	0.575

## Data Availability

You can get the public data used in this manuscript at TCGA (https://portal.gdc.cancer.gov/), CGGA (http://www.cgga.org.cn/), NCBI (https://www.ncbi.nlm.nih.gov/gds/?term=), and UCSC (https://xenabrowser.net/datapages/).

## Supplementary Materials

Supplementary Materials Supplement Figure 1: association of PLEKHA4 expression with clinical parameters. (a) disease stage, (b) gender, (c) age, and (d) 1p/19q code. ^∗^*p* < 0.05, ^∗∗^*p* < 0.01, and ^∗∗∗^*p* < 0.001; ns not significant. Supplement Figure 2: associations between the PLEKHA4. expression and immune infiltration level in LGG in CGGA and Rembrandt database. (a) The relationship between the expression of PLEKHA4 and the immune score of in CGGA LGG sample. (b) The relationship between the expression of PLEKHA4 and the immune score of in Rembrandt LGG sample. Supplement Figure 3: associations of the PLEHA4 expression level with lymphocytes, immunomodulators, and chemokines in LGG from TISIDB database. (a) Correlations between abundance of tumour-infiltrating lymphocytes (TILs) and PLEKHA4 (plus the six TILs with the highest correlation). (b–d) Correlations between immunomodulators and PLEKHA4 (plus the six immunomodulators with the highest correlation, respectively). (e, f) Correlations between chemokines (or receptors) and PLEKHA4 (plus the six chemokines (or receptors) with the highest correlation, respectively). Supplement Figure 4: survival difference between low and high-immune cell infiltration among PLEKHA4 high and low subgroups from Timer database, such as (a) CD4 T cells, (b) CD8 T cells, (c) M macrophages, and (d) B cells.
