# Supplementary material for: Overexpression of Pleckstrin Homology Domain-Containing Family A Member 4 Is Correlated with Poor Prognostic Outcomes and Immune Infiltration in Lower-Grade Glioma
Source: Dis Markers. 2022 Nov 11;2022:1292648. doi: 10.1155/2022/1292648 (PMC9674417; doi:10.1155/2022/1292648)
Supplement: Supplementary Materials — Supplement Figure 1: association of PLEKHA4 expression with clinical parameters. (a) disease stage, (b) gender, (c) age, and (d) 1p/19q code. ∗p < 0.05, ∗∗p < 0.01, and ∗∗∗p < 0.001; ns not significant. Supplement Figure 2: associations between the PLEKHA4. expression and immune infiltration level in LGG in CGGA and Rembrandt database. (a) The relationship between the expression of PLEKHA4 and the immune score of in CGGA LGG sample. (b) The relationship between the expression of PLEKHA4 and the immune score of in Rembrandt LGG sample. Supplement Figure 3: associations of the PLEHA4 expression level with lymphocytes, immunomodulators, and chemokines in LGG from TISIDB database. (a) Correlations between abundance of tumour-infiltrating lymphocytes (TILs) and PLEKHA4 (plus the six TILs with the highest correlation). (b–d) Correlations between immunomodulators and PLEKHA4 (plus the six immunomodulators with the highest correlation, respectively). (e, f) Correlations between chemokines (or receptors) and PLEKHA4 (plus the six chemokines (or receptors) with the highest correlation, respectively). Supplement Figure 4: survival difference between low and high-immune cell infiltration among PLEKHA4 high and low subgroups from Timer database, such as (a) CD4 T cells, (b) CD8 T cells, (c) M macrophages, and (d) B cells. [file 1292648.f1.pdf]

Supplement Figure1

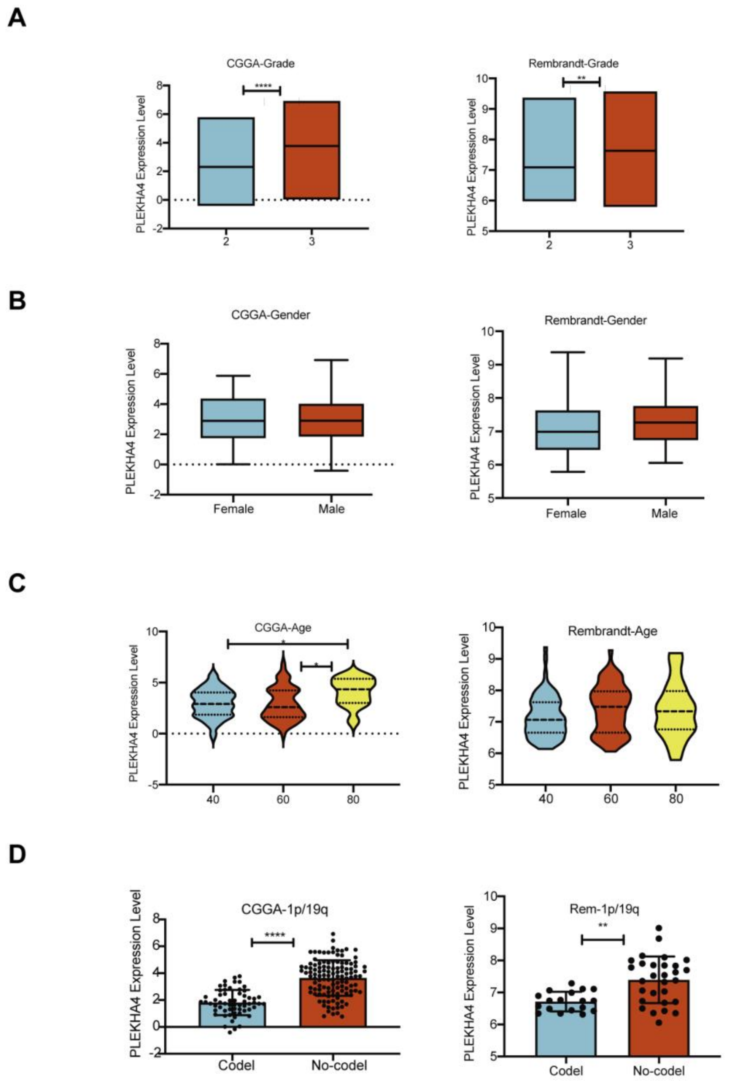

Supplement Figure 1. Association of PLEKHA4 expression with clinical parameters. (A) disease stage; (B) gender (C) age(D) 1p/19q code. *P < 0.05; **P < 0.01; ***P < 0.001; ns not significant.

Supplement Figure2

CGGA

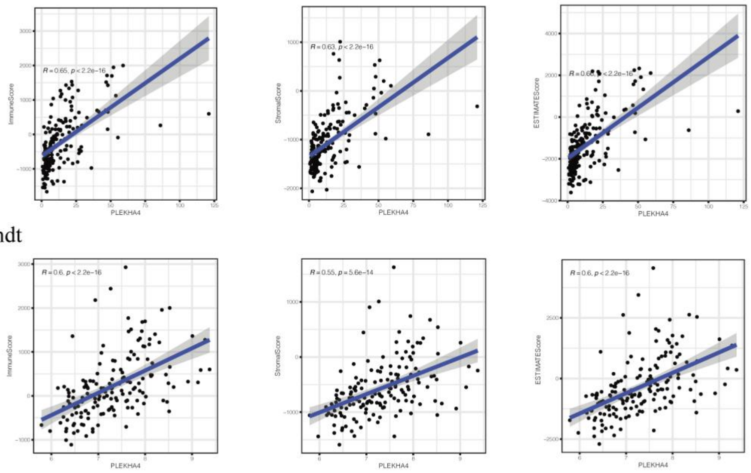

Supplement Figure 2. Associations between the PLEKHA4 expression and immune infiltration level in LGG in CGGA and Rembrandt database.(A) The relationship between the expression of PLEKHA4 and the immune score of in CGGA LGG sample(B) The relationship between the expression of PLEKHA4 and the immune score of in Rembrandt LGG sample

Supplement Figure3

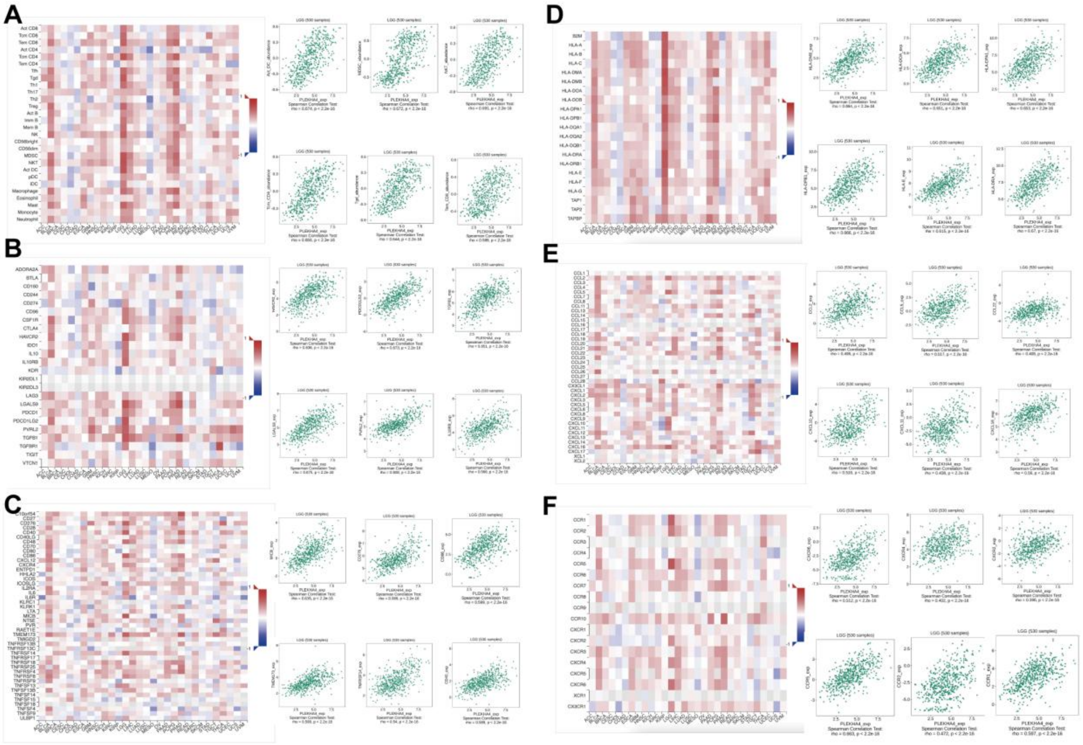

Supplement Figure 3. Associations of the PLEHA4 expression level with lymphocytes, immunomodulators and chemokines in LGG from TISIDB database. (A) Correlations between abundance of tumour-infiltrating lymphocytes (TILs) and PLEKHA4 (plus the six TILs with the highest correlation). (B-D) Correlations between immunomodulators and PLEKHA4(plus the six immunomodulators with the highest correlation, respectively). (E-F) Correlations between chemokines (or receptors) and PLEKHA4(plus the six chemokines (or receptors) with the highest correlation, respectively).

Supplement Figure4

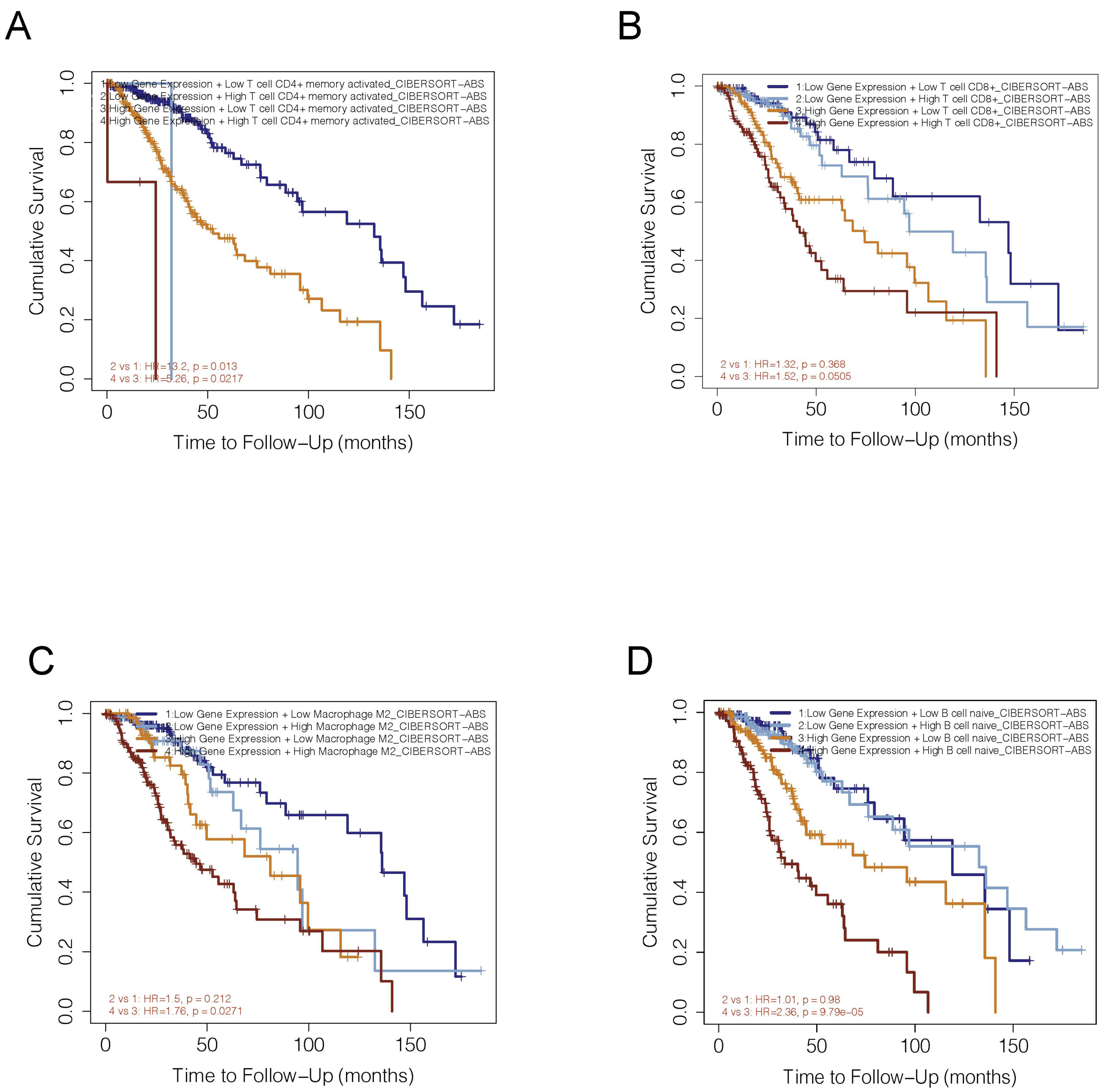

Supplement Figure 4.

Survival difference between low and high-immune cell infiltration among PLEKHA4 high and low subgroups from Timer database,such as CD4 T cells(A), CD8 T cells(B),M macrophages(C), and B cells(D).
